# 549 The Quality of TikTok Videos in the Education, Prevention, and Management of Burn Injuries

**DOI:** 10.1093/jbcr/irad045.146

**Published:** 2023-05-15

**Authors:** Sara Sheikh-Oleslami, Young Ji Tuen, Sally Hynes

**Affiliations:** University of British Columbia, Vancouver, British Columbia; Division of Plastic Surgery, University of British Columbia, Vancouver, British Columbia; BC Children's Hospital, Vancouver, British Columbia; University of British Columbia, Vancouver, British Columbia; Division of Plastic Surgery, University of British Columbia, Vancouver, British Columbia; BC Children's Hospital, Vancouver, British Columbia; University of British Columbia, Vancouver, British Columbia; Division of Plastic Surgery, University of British Columbia, Vancouver, British Columbia; BC Children's Hospital, Vancouver, British Columbia

## Abstract

**Introduction:**

Most burn injuries are preventable, and appropriate first aid post-injury results in improved outcomes for the patient as well as a reduced burden on the healthcare system. Many patients turn to internet resources for education and management approaches for their burn injuries. With the rising popularity of social media platforms for the promotion of health information, this creates a more accessible educational realm for those who may not know where to otherwise access informational resources. However, most content on social media is not validated or reliable. Previous studies have investigated the quality of online burn prevention, education, and management videos on platforms such as YouTube. In this study, we attempt to assess the quality of such videos on TikTok, a newer and rapidly growing platform.

**Methods:**

Videos on TikTok were searched for using 27 keywords (hashtags) such as #*burn, #education, #prevention, #management, *and #*firstaid*. The first 30 videos for each hashtag were reviewed. The Global Quality Scale (GQS) was used to assess the quality of the videos that fell within the inclusion criteria. Metrics such as views, viewer engagement, commentary, and likes were also examined.

**Results:**

Of 525 reviewed videos, 72 met inclusion criteria. 47.2% (n=34) were on first aid or medical management, 33.4% (n=31) were on basic information, and 9.7% (n=7) were on prevention. Only 5.6% (n=4) videos cited sources. The mean GQS score was 2.45. 50% of videos (n=36) were made by creators with medical training, and of those videos, the GQS score was 3.13, whereas content made by creators with no medical training had a mean GQS of 1.81 (*p *< 0.001). The excluded videos (n=453) fell into 6 main categories: unrelated (n=273), burn wounds (n=106), personal experiences (n=49), advertisements (n=8), questions (n=2), and non-English videos (n=15). Viewership varied from 41 to 4.2 million views.

**Conclusions:**

Overall, information regarding burns on TikTok is unsatisfactory. Additionally, viewers are more drawn to lower quality videos. While quality varies amongst the different sources and is dependent on the creator, there is a significant gap in reliable content regarding burns, demonstrating an opportunity for validated content creation in this realm. Patients must exert caution and be selective when using TikTok as an educational resource regarding burns.

**Applicability of Research to Practice:**

This review addresses key gaps in the knowledge base on TikTok for reliable burn information. This will later be used as a framework to create a series of educational videos for patients.

